# Schistosomiasis Morbidity Hotspots: Roles of the Human Host, the Parasite and Their Interface in the Development of Severe Morbidity

**DOI:** 10.3389/fimmu.2021.635869

**Published:** 2021-03-12

**Authors:** Patrice A. Mawa, Julien Kincaid-Smith, Edridah M. Tukahebwa, Joanne P. Webster, Shona Wilson

**Affiliations:** ^1^ Immunomodulation and Vaccines Programme, Medical Research Council-Uganda Virus Research Institute and London School of Hygiene and Tropical Medicine (MRC/UVRI and LSHTM) Uganda Research Unit, Entebbe, Uganda; ^2^ Department of Immunology, Uganda Virus Research Institute, Entebbe, Uganda; ^3^ Department of Infection Biology, London School of Hygiene and Tropical Medicine, London, United Kingdom; ^4^ Centre for Emerging, Endemic and Exotic Diseases (CEEED), Department of Pathobiology and Population Sciences (PPS), Royal Veterinary College, University of London, Herts, United Kingdom; ^5^ Vector Control Division, Ministry of Health, Kampala, Uganda; ^6^ Department of Pathology, University of Cambridge, Cambridge, United Kingdom

**Keywords:** schistosomiasis, biological hotspot, morbidity, host-parasite-environmental-factors, FibroScHot

## Abstract

Schistosomiasis is the second most important human parasitic disease in terms of socioeconomic impact, causing great morbidity and mortality, predominantly across the African continent. For intestinal schistosomiasis, severe morbidity manifests as periportal fibrosis (PPF) in which large tracts of macro-fibrosis of the liver, visible by ultrasound, can occlude the main portal vein leading to portal hypertension (PHT), sequelae such as ascites and collateral vasculature, and ultimately fatalities. For urogenital schistosomiasis, severe morbidity manifests as pathology throughout the urinary system and genitals, and is a definitive cause of squamous cell bladder carcinoma. Preventative chemotherapy (PC) programmes, delivered through mass drug administration (MDA) of praziquantel (PZQ), have been at the forefront of schistosomiasis control programmes in sub-Saharan Africa since their commencement in Uganda in 2003. However, despite many successes, ‘biological hotspots’ (as distinct from ‘operational hotspots’) of both persistent high transmission and morbidity remain. In some areas, this failure to gain control of schistosomiasis has devastating consequences, with not only persistently high infection intensities, but both “subtle” and severe morbidity remaining prevalent. These hotspots highlight the requirement to revisit research into severe morbidity and its mechanisms, a topic that has been out of favor during times of PC implementation. Indeed, the focality and spatially-structured epidemiology of schistosomiasis, its transmission persistence and the morbidity induced, has long suggested that gene-environmental-interactions playing out at the host-parasite interface are crucial. Here we review evidence of potential unique parasite factors, host factors, and their gene-environmental interactions in terms of explaining differential morbidity profiles in the human host. We then take the situation of schistosomiasis mansoni within the Albertine region of Uganda as a case study in terms of elucidating the factors behind the severe morbidity observed and the avenues and directions for future research currently underway within a new research and clinical trial programme (FibroScHot).

## Introduction

Species of the genus *Schistosoma* are digenetic trematodes and the causative agents of the Neglected Tropical Disease (NTD) schistosomiasis; a parasitic disease that ranks second only to malaria in terms of socioeconomic impacts. Over 220 million people worldwide are currently infected, 90% of whom live in sub-Saharan Africa (SSA) (1, 2), with an estimated annual mortality of at least 200,000 (3). Infection in humans, as well as alternative mammalian definitive hosts, occurs in contaminated freshwater environments *via* cercariae shed from specific snail intermediate hosts. Early acute morbidity can occur following cutaneous penetration, sometimes leading to an urticarial rash known as swimmers itch or cercarial dermatitis (4, 5). After entering the host, schistosomes migrate through the body to mature to adulthood in the liver. This phase can also involve a second major acute stage around four weeks post exposure, known as Katayama fever, typically characterized by fever, urticarial rash, enlarged liver and spleen and bronchospasm (6). The precise pathogenesis of Katayama fever is unknown, and is also frequently missed from diagnosis, hence little is known about potential differential morbidity profiles by parasite species, host or habitat - although it is suspected to involve an immune complex phenomenon initiated by the maturing schistosomes and potentially their eggs. The major chronic clinical manifestations of human schistosomiasis are, however, primarily associated with the species-specific oviposition site of the adult schistosomes. With the female residing, and maturing, within the gynecophoral canal of the larger male, schistosome males carry the pair to the mesenteric plexus (for the intestinal schistosome species – predominantly *S. mansoni* within SSA, South America, the Caribbean and the Yemen, with *S. intercalatum* and *S. guineensis* as minor species within Africa, and *S. japonicum* or *S. mekongi* across parts of Asia) or to the veins of the pelvis (for the urogenital species *S. haematobium*). Sexual reproduction [and in certain cases parthenogenesis (7)] between the gonochoric adults results in the production of up to hundreds or thousands of eggs per day per female worm, depending on the species (on average, 20-200 for *S. haematobium* (and potentially hybrids therein), 100-300 for *S. mansoni*, but 500-3000 for *S. japonicum)*. Only a proportion of these spined eggs are excreted *via* the host’s feces in the case of intestinal schistosomiasis or urine in urogenital schistosomiasis to pursue the parasite’s life cycle. The remainder of the eggs remain trapped in the host tissues inducing granulomatous and fibrotic responses *via* the host’s immune system (8). In children, continued inflammation has been reported to impede, amongst other pathologies, iron metabolism, with consequent disabling systemic morbidities including anemia, malnutrition, physical fitness and impaired physical and cognitive development (9, 10).

For urogenital schistosomiasis, long-term chronic infection may lead to lesions in the vesical and ureteral walls, resulting in fibrosis of the bladder and lower ureters, calcification of the urinary tract, and kidney dysfunctions. This chronic inflammation of the bladder can ultimately cause squamous cell bladder cancer (11). Lesions of both the male and female genital tracts, known as genital schistosomiasis, are understood to be potential causes of sterility and maternal fatal hemorrhaging during child birth, as well as risk co-factors for sexually-transmitted diseases such as HIV (11–13). Chronic intestinal schistosomiasis, in its severest form, can cause the development of periportal fibrosis (PPF), characterized by large tracts of fibrotic material laid down along the liver vasculature. With PPF, blood flow through the liver can become restricted leading to the development of portal hypertension, associated collateral vasculature and esophageal varices, and often accompanying ascites. Death can occur through hematemesis caused by the rupturing of esophageal varices (14, 15).

Sub-Saharan Africa currently carries the major global burden of schistosomiasis, and hence, since 2003, large-scale mass drug administration (MDA) programmes of praziquantel (PZQ), as preventative chemotherapy (PC), has been implemented across much of SSA (16). Morbidity control has been, in many countries, generally successful (17) and this helped lead to a revision of the World Health Organization (WHO) strategic plan for a vision of “a world free of schistosomiasis”, which included controlling morbidity of schistosomiasis by 2020 (defined as prevalence of heavy-intensity infection <5% aggregated *across* sentinel sites) (17, 18). Likewise, the newly-launched revised WHO 2021-2030 NTD Roadmap, aims to eliminate schistosomiasis as a public health problem (EPHP, defined as elimination of morbidity where prevalence of heavy infection intensity is less than 1% in all sentinel sites) in all endemic countries by 2030 (19). Complete interruption of transmission (reduction of incidence of infection to zero) is also a target in selected African regions by 2030 (19). Likewise, China has set the target of reaching complete interruption of transmission at the country level by 2030 (20). However, recent reports of schistosomiasis being more prevalent than previously thought (21), indications of potentially reduced drug efficacy among populations under high MDA pressure (22, 23), continued persistent and/or re-emerging ‘hotspots’ of infection (encompassing both operational and biological hotspots), in terms of infection prevalence (17, 24), intensities (25–32) and/or severe host morbidity (33–37) all serve to highlight that schistosomes are highly complex multi-host parasitic organisms for which many essential characteristics of their biology and epidemiology, and the human host response to exposure and infection, remain largely unknown.

One long-standing unanswered question in disease epidemiology in general is why pathogen/parasite prevalence, intensity and associated morbidity are heterogonous across space and time and what factors may be underlying these differences. Host-parasite interactions involve a complex co-evolutionary interplay, where antagonist factors from the host (i.e., defense mechanisms) and the parasite (i.e., infective and/or virulence strategies) play a significant role in determining the outcome in terms of both transmission potential and pathology induced. While host factors and environmental differences have been at the center of most studies trying to understand these disparities, parasite factors, in comparison, have been overlooked. This scenario is certainly true for schistosomiasis for which a key epidemiological feature is that parasite factors are not uniform across space and time. There is substantial heterogeneity in terms of infection prevalence and/or intensity among different geographic areas and individual hosts, even within relatively close locations of the same region (38–41). Likewise, studies have revealed that the disease severity can vary considerably at both the geographical and individual level for both *S. mansoni* (42–45) and *S. haematobium* (46–48), associated in part, but not always, in focality to accompanying high transmission and infection intensities (39, 45, 49–52). Epidemiological evidence suggests such variation in morbidity could also be partially explained by, among other factors, the immunological and genetic background of the endemic communities, their nutritional status and/or the length of time individuals have been exposed (45, 53–57). However, only a few studies have aimed to assess if potential parasites factors may contribute to this dichotomy in morbidity and particularly in PPF and its complications. In line with the objectives of schistosomiasis control, and the EPHP targets in particular, elucidating the relative etiologies behind such geographical and individual variations in morbidity are of profound importance. Here we review the potential unique parasite factors, human host response factors, and their gene-environment interactions in terms of explaining persisting morbidity hotspots. We then take the situation of schistosomiasis mansoni within the Albertine region of Uganda as a case study in terms of elucidating the potential factors behind the severe morbidity observed to date and the avenues and direction for research currently underway within a new clinical trial programme (FibroScHot).

## The Role of the Parasite in Morbidity Hotspots

By definition parasites are harmful to their hosts, and at its simplest, schistosomiasis-associated morbidity levels have been associated with infection intensity. The recent expansion of MDA programmes with PZQ has led, in general, to significant reductions in schistosomiasis prevalence, intensity and subsequent human host morbidity (17, 37, 58). However, it remains to be ascertained how such MDA, together with other anthropogenic selection pressures imposed by our changing world, may impact upon parasite fitness and strategies, nor how this in turn may affect their genetic diversity, transmission dynamics, virulence, clinical outcome and drug resistance development across Africa (59). Early studies in the laboratory have clearly shown that schistosomes with reduced susceptibility to PZQ can be selected for (60, 61), but that this resistance comes with a cost in terms of reduced schistosomes reproductive fitness (62) as well as genetic diversity (63–65). Furthermore, within only a few generations, selective pressures imposed on laboratory-bred schistosomes can produce rapid changes in life history traits including parasites infectivity, fecundity, transmission and virulence (66–74). Likewise, field-based studies in which implementation of large-scale intervention trials on optimal treatment in various zones of Africa, have shown a strong variability of response to annual MDA (30). For example, certain villages in the Nile Delta remain highly prevalent to *S. mansoni* despite over two decades of MDA (75). Other locations such as in Côte d’Ivoire have shown an initial decrease in *S. mansoni* infections after MDA sometimes followed by an increase in prevalence the following years (76). Moreover, after multiple rounds of MDA in the transmission hotspots of Mayuge District, Uganda, egg reduction rates (ERR) were found to be reduced to below the WHO recommendation of 90% in contrast to that observed amongst school-children in similar regions but with a lower past MDA pressure history (22). Assessing therapeutic efficacy of PZQ against schistosomes and the changes in parasites’ susceptibility is thus particularly important (22, 23). Also in Uganda, the communities on the shores of Lake Albert are a typical example of how despite strong efforts to lower the burden of high infection intensities PPF can remain common (33, 35, 77–79). Such locations where *Schistosoma* spp. infection fails to decline in prevalence and/or intensity to expected levels despite multiple years of annual MDA, in comparison to locations that simply have high prevalence before intervention, can be considered as persistent “hotspots” (25–27, 80). As drug resistance is commonly associated with life-history costs (81–84), the potential for drug resistance and associated trade-offs may be important factors in the maintenance of high infection intensities and morbidity levels across Africa. The success, or not, of control strategies in several endemic areas is thus likely to be affected by host-parasite-drug interactions and these associated trade-offs have raised concerns there may be reduced drug efficacy, especially in communities with a more intensive history of PZQ treatment (22, 85, 86). However, while some degree of reduced drug susceptibility has been suggested (22, 86–90), more data are warranted to clarify the evolution of such responses under field conditions and dissect potential resistance from, for example, differential host clearance responses and/or rapid reinfection that seems to best explain apparent low cure rates in most situations (88).

Additional to potential PZQ resistance, other notable parasite factors may contribute in maintaining high egg outputs and hence potential high host morbidity, despite efficient MDA coverage. Among these factors, density-dependent fecundity compensation, which is a common feature of several microparasite and macroparasite (notably helminths) life cycles may regulate the parasite reproduction and transmission dynamics in a way that make the worm-egg relationship non-linear (19) and in some cases geographically variable (91). Density-dependent fecundity can either be positive at low levels of infection (e.g. density-dependent facilitation) or negative at high parasite densities (e.g. density-dependent inhibition) because of intra-host competition for resources and/or immunological host responses (92, 93). Although still controversial, studies on *Schistosoma* worm burdens and associated egg counts have shown potential evidence for density-dependent fecundity inhibition in the two major human infecting species, *S. mansoni* and *S. haematobium.* The precise nature of the phenomenon is not well understood, since the only direct data that exists is from a limited number autopsy studies (94) and with subsequent biostatistical debate over their original conclusions (94–96). Empirically, population genetic analyses including parentage analysis to estimate the adult worm burden performed on data from Mali (15, 97) and Tanzania (98–102) have shown that despite no evidence of a reduction in mean infection intensity (e.g. egg counts) by the standard parasitological techniques (e.g., Kato-Katz), the worm burdens were declining within individual hosts after PZQ administration, thus underlying a relaxation of the density-dependent fecundity inhibition among *S. mansoni* populations (103). However, comparison of cross-sectional levels of the adult worm derived cationic anodic antigen (an indirect measure of worm burden), against egg counts have shown a diversion from the linear between the measurement, particularly in older individuals, for *S. haematobium* (104, 105) but not *S. mansoni* for which a linear relationship has been reported (104). These observations raise fundamental questions on schistosome population biology and strongly suggests that, despite a reduction in the adult worm population after treatment, such parasites factors may contribute to population persistence and resilience by producing characteristic epidemiological patterns maintaining the global infection intensities and morbidity to unexpectedly high levels (106, 107). Importantly, increasing the reproduction rates of parasites that survive treatment and potentially harbor drug-resistant or virulence related alleles may be expected to increase the spread of such traits in the populations (106, 107). Finally, by shaping the transmission dynamics of the parasite and its potential response or resilience to control measures, such processes may complicate both the monitoring and implementation of chemotherapy (103, 107, 108).

Another critical biological parasite factor for consideration is that, whilst disease control programmes, at least in terms of their monitoring and evaluation, tend to consider schistosome-specific morbidity in isolation, parasites under natural situations do not exist in isolation. Inter-genera, inter-specific and even intra-specific interactions are likely and, in many cases, may be predicted to differentially impact the morbidity inflicted upon their hosts *via* antagonistic or synergistic effects (109, 110). For example, across much of SSA, mixed species infections of both *S. mansoni* and *S. haematobium* are common (111–115). Such co-infections may lead to co-morbidities with pathological symptoms being a combination between those of the parasite species. A series of studies across Cameroon, Niger, Kenya and Senegal have found that mixed *S. mansoni* and *S. haematobium* infections decrease hepato-splenic morbidity compared to single *S. mansoni* infections and increase urogenital morbidity compared to single *S. haematobium* infections (111, 116, 117). The lowering effect observed on liver morbidity is believed to be because dominant *S. haematobium* males divert *S. mansoni* females from the portal vein to the vesical plexus, resulting in more eggs being passed through the urogenital tract and less eggs being delivered to the liver tissues. Similarly, in their recent study Huyse and colleagues showed an intriguing association between *S. mansoni* genetic variation and bladder morbidity (118), again potentially explicable through *S. mansoni* females being paired with *S. haematobium* males that guide females towards the urogenital system (118). In Senegal, ectopic elimination of eggs is also common (39, 89, 119–121). Distinct studies undertaken there showed that urine samples from people with mixed infections of *S. mansoni* and *S. haematobium* contained 31% (119) and 13% (121) of *S. mansoni* eggs. In the latter study people infected with both species and eliminating *S. mansoni* eggs both *via* urine and *via* stool had the highest risk of bladder morbidity (121). Likewise, on a same note, Ernould and Sellin found cure rates to be much lower in the Senegalese village with mixed infection compared to villages with single infections (119). The authors found that after treatment S*. haematobium* infection remained low, whereas egg excretion by *S. mansoni* was seven times higher than at the start of the study. The authors argued that in addition to the possibility of PZQ resistance, or as more likely, potentially higher force of infection/rapid re-infection of *S. mansoni* from the environment at this point, relative to *S. haematobium*, the elimination of *S. haematobium* after treatment and heterologous pairings allowed remaining *S. mansoni* females to pair with *S. mansoni* males that survived treatment.

Furthermore, with gathering development in molecular typing, and potentially in line with ongoing major anthropogenic changes in the environment, people across large expanses of, particularly Western, Eastern and Southern SSA, have been found to be infected with viable hybridized schistosomes involving the human urogenital *S. haematobium* with the intestinal schistosome species of livestock *S. bovis*, *S. curassoni*, *S. mattehii* and beyond (21, 122–127). Given the pairings of urogenital with intestinal schistosome species here, we may well predict similarly differential morbidity profiles as that observed for the aforementioned *S. mansoni* with *S. haematobium* (114, 128). Indeed, given that these Haematobium group hybrids produce viable eggs, in contrast to the more phylogenetically distant *S. mansoni* with *S. haematobium* pairings, one may predict exacerbated differentiating morbidity profiles in relation to infection status (129). Hybridization between genetically distinct species raises the possibility of promoting genetic admixture and diversity, introducing novel genes across species boundaries, but also lead to the emergence of novel hybrid zoonotic strains with an increased transmission potential that could have serious implications for the control of the disease (124, 129–131). The differential impact of such inter-specific interactions on the host morbidity profiles observed are pertinent in terms of highlighting the need to, wherever possible, measure both hepatic and urogenital morbidity indicators during MDA monitoring and evaluation where co-infections and zoonotic species are known or suspected to exist.

The role of intra-specific differences and interactions on the host morbidity profile must also be considered (132, 133). Although the role of the parasite genetic diversity in differential host response is well known for microparasites (134, 135), there is comparably less known regarding the potential importance of macroparasite genetic heterogeneities in general, and schistosomes in particular, on disease epidemiology (136). Laboratory studies, however, have shown that schistosomes strains or populations from the same or different geographical locations can show a number of differences in biological traits related to transmission and virulence such as infectivity, egg production, pathogenicity and response to chemotherapy (11, 137–146). For example, some *S. haematobium* strains studied in the laboratory show different levels of mortality and worm recovery in hamsters as well as differences in snail infectivity (147), while different *S. mansoni* strains have been shown to induce disparate rates of hepatomegaly and splenomegaly despite comparable fecal egg counts (137). Moreover, alternative transmission strategies with the occurrence of trade-offs between parasite transmission and host survival have been observed in genetically different schistosome populations (148). Hence, this shows that schistosomes’ virulence occurs with significant variation for both intermediate and definitive hosts on a genotype-dependent basis, demonstrating that virulence and transmission may vary across individuals of a population and/or between populations (66, 140, 148).

Species–specific microsatellite markers developed for *S. mansoni* (149–152) and *S. haematobium* (103, 153) have also provided a better understanding of schistosomes epidemiology and transmission patterns through investigation of genotypic associations at the population level in the field (154). Such studies have revealed considerable variation in schistosome populations with high levels of genetic diversity mainly finding its origin at the infrapopulation level (41, 152, 155–163). Schistosomes are mostly structured according to distance between transmission sites with limited gene flow at both a regional and continental scale (65, 157, 164). However, patterns of population structure vary between different regions and epidemiological settings (165) and while some studies show that the parasite’s genetic variation is usually randomly distributed at relatively small scales with high levels of gene flow within and between hosts and sites (89, 154, 156, 163, 166, 167), others show that some populations may be significantly differentiated even among relatively close geographic locations (41, 160, 168, 169). In Uganda, for example, evidence suggests that parasite population genetics are potentially playing a role in the variations in morbidity found between Lake Albert and Lake Victoria. In addition to disparate levels of morbidity with higher levels of PPF in Lake Albert communities, previous barcoding of *S. mansoni* collected from both definitive human hosts and intermediate snail hosts on the shores of Lake Albert in Uganda and Lake Victoria in Kenya, Tanzania and Uganda revealed that the population genetic structure of *S. mansoni* is not uniform across the endemic area. Whilst both populations are extensively diverse, studies showed that in Lake Victoria non-synonymous mutations were more diverse than in Lake Albert and that there was a strong genetic differentiation between the two parasite populations (167, 169–173). Interestingly, other studies have shown that parasites from Lake Victoria area present different local strains (173), with the relatively highest levels of genetic diversity across several markers (22, 155, 157, 174). The epidemiological heterogeneity of intestinal schistosomiasis between these lake environments could thus be due to parasite diversity itself (117, 172) and the lack of gene flow strongly suggests that any locally evolved traits, such as virulence or putative drug resistance would likely stay restricted to the focal population, leading, at least in part, to differential host morbidity. Previous studies conducted in Mali, Senegal and Uganda have, however, found no associations between infection intensity and parasites genetic diversity when comparing allelic richness, heterozygosity, nor parental genotypes to various levels of infection intensity (103, 156, 167). Nonetheless, the parasite’s virulence measured through its fecundity could in part be linked to the parasite’s genetic diversity or associated to a particular genotype. Although few potential direct links between parasite genetics and host induced pathology are to yet be made, various authors have suggested that schistosomes infection intensity and the parasite populations’ genetics in different African countries may be responsible for such discrepancies in clinical outcomes by acting on several parasite features including their fecundity and immunogenicity (53, 132, 166, 175, 176). Nevertheless, our knowledge on the role of parasite genetic variation in host disease phenotype in human schistosomiasis is currently limited and only few studies have directly investigated the relationship between morbidity and the genetic variation.

Brouwer and colleagues (133) gave the first insight in this delicate host-morbidity/parasite-genetics association by focusing on *S. haematobium*, the species responsible for urogenital schistosomiasis. Using randomly amplified polymorphic DNA (RAPD) between *S. haematobium* populations from children with varying pathology of urinary tract in Zimbabwe the authors compared the distribution of *S. haematobium* genotypes in the definitive host in relation to that of the clinical outcome (133). They showed that the allelic frequencies at eight loci differed significantly between the mild and severe groups and that three clusters were significantly over-represented in schoolchildren with severe urogenital lesions. Inspection of allelic distributions for clusters revealed that cluster 1 (severe) and cluster 7 (mild) had inverse genotypes at loci that differed significantly between groups, supporting the notion that particular parasite strains or genetic factors may be associated with clinical outcome. However, they could not robustly link pathology to parasite genotypes or genes due to the limitations of the RAPD technique. Further studies were therefore conducted on *S. haematobium* with the aim of elucidating any potential relationship between host morbidity and parasites genetic variation. In a second study, while the authors found high levels of genetic diversity among the three isolates studied (Egypt, Zimbabwe, and South Africa) they did not identify a role of parasites genetic diversity in the difference in morbidity observed (132). Finally, in a third study, the same authors used the RAPD based-approach in Sudan, but found no association between abnormal ultrasound urinary tract scans and intensity of infection (177) nor could they identify any significant difference when comparing the three different genotypes identified with the severity of the disease (178). However, the authors suggested that differences in parasite strains, such as infection intensity, could partially explain why they failed to retrieve similar results to those previously observed by Brouwer in Zimbabwe (133), together with the small number of variable alleles recorded in their study largely hampering their ability to detect associations between diversity and host morbidity.

In *S. mansoni*, at least three studies have been conducted on host characteristics and putative parasite genetic variation. In the first one, Barbosa and colleagues (179) used 15 microsatellite markers and found geographic clustering in *S. mansoni* over a scale of 8 km. However, the authors could not link this with demographic or epidemiological host characteristics (179). A second study using 11 microsatellite markers (41), suggested the existence of a link between parasite genetic diversity and prevalence and intensity of infection between three settings. The authors observed that the gradient they found in genetic diversity was the same as the gradient they previously observed when focusing on the parasite prevalence and intensities of infection. However, because only three populations were sampled, they could not statistically validate this link between parasite genetic diversity and parasite virulence (41). Finally, Huyse and colleagues are to date the only ones to have formally demonstrated a potential link between the parasite genetic variation and host disease phenotype in humans (118). Using nine microsatellite markers on 1561 *S. mansoni* larvae collected from 44 human hosts in Senegal they were able to link host characteristics such as age, sex, infection intensity, liver and bladder morbidity to the parasites genotypes. They showed a significant association between allelic variation at the parasite locus L46951 and host infection intensity and morbidity. This locus is located near a gene (cGMP-dependent protein kinase) linked to schistosomes egg production. Furthermore, by reconstructing the parental genotypes the authors suggest that adult parasite populations with the allele L46951 have a higher fecundity and therefore produce more eggs and offspring than those without this allele, thus potentially inducing higher levels of morbidity (118).

Despite scarce studies and some contradictory results, or at least issues in the methodology and detection power hampering the authors’ ability to link parasites genotypes with host characteristics, these studies clearly highlight the importance of genetic variation as an additional factor to schistosomiasis host disease phenotypes, including a potential association with persistent hotspots. It is necessary to take into consideration parasite genetics and population diversity in future epidemiological studies to make a clear relation between transmission and morbidity in different geographical zones where particular parasite genotypes may be predicted to interact differentially with their host and lead to differences in morbidity. One of our greatest limitations to date is the absence of genetic markers sufficiently powerful to accurately allow us to identify and link regions involved in the parasite’s virulence or fecundity with host morbidity indicators. Such data may be valuable in monitoring relationships between the parasite’s transmission (prevalence and intensity) and virulence with particular parasite genotypes or degrees of genetic diversity in clinical phenotypes. Nevertheless, since microsatellites are neutral markers, they are not expected to identify such parasites traits unless they are physically close to such genes. One potential first step would thus be to properly define and find a consensus on what should be considered as parasite virulence factors in schistosomes and other macroparasites of medical and/or veterinary interest, while a second fundamental step would be the development of specific virulence/fecundity markers or whole genome Single Nucleotides Polymorphism (SNP) markers allowing us to address the challenges at the interface between parasite genetic factors and host induced morbidity.

## The Role of the Host in Morbidity Hotspots

As stated above, a key epidemiological feature of schistosome infection is that the parasite prevalence, infection intensity and associated morbidity are not uniform across space and time, with substantial heterogeneity among different geographic areas and individual hosts even within relatively close locations of the same region (40, 41). In addition, familial aggregation suggests that host-intrinsic and not just behavioral factors can be involved in development of severe morbidity in humans (180, 181). There have been a number of studies convincingly demonstrating that genetic factors are important within intermediate snail hosts in terms of their susceptibility and/or subsequent morbidity to schistosomiasis and in promoting heterogeneity in patterns of infection (see (71) and (182, 183) for review). In Uganda, for example, a fundamental factor of differing snail host populations could drive differing transmission dynamics of *S. mansoni* between Lake Albert and Lake Victoria, with prevalence levels and reinfection rates higher at Lake Albert (170, 184). Indeed *Biomphalaria stanleyi* is found only in Lake Albert, and *B. choanomphala* is present only in Lake Victoria, while *B. sudanica and B. pfeifferi* are present in both lakes (185–189). Such intermediate host–specific factors could have influenced the evolutionary history of the parasites, playing an important role shaping the genetic composition of schistosome populations (156, 174, 190, 191) and selecting for particular parasite genotypes of varying virulence.

Direct evidence from the definitive hosts, and the human host in particular, is in contrast less available due, in part, to an inherent inability to perform controlled studies. Our knowledge of the host intrinsic (rather than behavioral factors) underlying disease dynamics and mechanism are therefore accumulated from autopsy and observational epidemiological studies that are clinical, genetic or immunological in design; combined with animal experimental models for which the evidence is heavily skewed towards *S. mansoni* due to the existence of a well-established murine model for this infection. The mouse is not permissive to *S. haematobium*, with hamsters being utilized for lifecycle maintenance. This, coupled with a differing *S. haematobium* predilection site within the hamster (the blood supply of the intestine, rather than of the venus-plexus of the pelvis as in humans) has resulted, until the introduction of a murine egg micro-injection experimental model (192, 193), in a relative scarcity of evidence for the immunopathological mechanism in *S. haematobium* infection. Without knowledge of the underlying mechanisms of severe morbidity, relating variation in host factors across a geographical scale to indicate the presence of morbidity hotspots is not possible.

Our early understanding of much of the clinical syndrome of human intestinal schistosomiasis was gained through the ground breaking autopsy work conducted by Alan Cheever and colleagues in the 1960s in Brazil. Due to the host blood flow, in *S. mansoni* infection a large proportion of the eggs laid by the female worms get trapped in the distal site of the liver. In all cases of liver PPF observed by Cheever (n=105), infection with *S. mansoni* was present, and amongst the cases 85% had varices, and hematemesis through rupturing of esophageal varices was the main cause of death (14); thus establishing *S. mansoni* as the causative agent of PPF and its consequences. In addition, after perfusion of the cadavers he concluded that worm burden was positively associated with presentation with PPF and its complications (15). Cheever did, however, warn that any observations made within his autopsy studies may not reflect the epidemiological patterns observed within populations of endemic areas as the demographic profile of autopsy cohorts are by their inherent nature skewed towards the older sections of the human population and/or those with severe disease who died young. Repeat autopsy studies conducted in Egypt where *S. mansoni* and *S. haematobium* are endemic confirmed the linkage between PPF and intestinal schistosomiasis through presence of *S. mansoni* eggs within macroscopic PPF lesions of the liver, and discounted a link with *S. haematobium* (194).

At the time of the autopsy studies, clinical palpation of the liver and spleen were the standard method of assessing whether an individual had severe schistosomiasis. It was therefore not until the 1980s and the introduction of portable ultrasound machines that researchers were able to establish the true relationship between presentation with PPF and demographic and infection related parameters. A dissociation between peak infection levels and peak prevalence of PPF was observed, with fibrosis more apparent in adults than in adolescents, the age group who carry the greatest infection burden (53, 195, 196). Duration of ongoing infection, as well as high transmission levels, are now known to be important in the development of PPF and its severity upon assessment (33). As adults, particularly males, are more likely to have severe PPF (53), and this is linked more to past exposure than current infection, treatment studies with PZQ, which reduces infection burden but does not directly treat the fibrosis, have shown that regression of PPF is less likely to be observed amongst this demographic (197–199). In children, on the other hand, PPF is often mild, but clearly observable by ultrasound, and can respond well to treatment. Amongst children regression of PPF can be observed through a reduction in severity score at 7-months post-treatment (200), with full resolution being observable by 2-years post-treatment (201). However, the success of treatment of PPF is also dependent on the force of transmission, with the rapid re-infection that can occur in high transmission areas impeding the success of treatment (199).

In contrast to *S. mansoni* where severe morbidity occurs in tissues distal to the site of predilection, in *S. haematobium* the immediate sites of egg deposition are those associated with severe morbidity. *S. haematobium* worms pairs are believed to be relatively sedentary, so the resulting tissue inflammation is very focal, leading to bladder wall thickening in areas where eggs are deposited regularly (97, 202). It has been proposed that there is no pattern to where in the bladder the lesions occur (203). Interaction between responding immune cells and the neighboring urothelial cells causes urothelial hyperplasia (202), which, with time, can result in the development of ultrasound detectable masses (defined as >1cm thickening of the bladder wall) and pseudopolyps (204). Younger children mostly have what has been defined as milder bladder pathology, characterized by wall thickening or irregularities (205). These irregularities are likely to represent early polypoid lesions, which in autopsy studies were observed around live eggs so indicative of active infections prior to the death of the younger cadavers (202). In epidemiological studies, a strong predictor (though not a conclusive marker) of bladder morbidity, particularly in children, is infection intensity (206–208). These bladder wall irregularities therefore resolve in a manner similar to, but more readily than the resolution of mild PPF after treatment with PZQ, with full resolution observable within 6-months (205, 209, 210). However, again similar to *S. mansoni* associated morbidity, in high transmission areas the success of a single round of treatment is impeded by rapid re-infection and the associated re-emergence of ultrasound detectable morbidity within a year (205, 209, 210). In contrast, upper urinary tract morbidity resolution post treatment is less successful (205).

The immuno-pathology secondary to schistosome infection is thought to be as a result of host immune responses to egg antigens rather than direct damage to the tissues by the eggs. It is thought in *S. mansoni* infection that the arising tight granuloma formation, characterized by concentric rings of encapsulating immune cells around the eggs and subsequent collagen deposition, may lead to advanced hepatosplenic schistosomiasis (1, 11, 143, 211). Murine studies show that granulomas formed during *S. mansoni* infection are as a result of T helper (Th) 2 cytokines produced by CD4^+^ T cells (212). This results in the recruitment of immune cells including eosinophils, monocytes, alternatively activated macrophages, basophils, T and B cells to the site of inflammation (213), and is aimed at containing the eggs and their hepatotoxic products (214–216). How these early granuloma responses relate to the long-term consequence of PPF observed within humans with intestinal schistosomiasis remains largely unknown. In addition, despite the egg micro-injection murine model of *S. haematobium* morbidity indicating similar granuloma formation in response to the eggs of this species, there are very significant biological differences between *S. haematobium* and *S. mansoni*, including the absence of a *S. haematobium* Ω-1 homologue (216), a major immunogenic glycoprotein secreted by *S. mansoni* eggs that drives Th2 polarization by dendritic cells (217, 218). The experimental data from *S. mansoni* egg immuno-biology is therefore not directly transferable to *S. haematobium* eggs.

At the human host genetic level, polymorphisms in the interleukin (IL) -13 gene have been associated with susceptibility to *S. haematobium* infection (219) and the loci of the IL-13 gene, 5q31-q33, has been associated with susceptibility to *S. mansoni* infection (220). However, care must be taken in differentiating a type-2 response associated with protection from infection *per se*, which is more significantly linked with immune responses to adult worm derived antigens ( (221) for review), from those responses that arise to egg antigens and cause morbidity. That said, in line with the evidence from murine studies that Th2 cytokines produced by CD4^+^ T cells induce granuloma formation, human clinical-immunological studies have found PPF to be associated with sustained Th2 responses (222). One suggested mechanism for fibrosis from non-schistosome human studies is the Th2 cytokine-induced differentiation of CD14^+^ monocytes into fibrocytes (223). Whether this is occurring in the context of schistosome infection is not known. In humans, we have shown that Th2 responses to *S. mansoni* egg antigens in adults from a fishing village in Uganda (224) and school-aged children in Kenya (225) are generally suppressed during active *S. mansoni* infection. A study by Colley et al. (226) suggests that dysregulation of T-cell responses may be causative of PPF. However, this study was based on T cell expansion assays and does not mention the type of CD4^+^ T cells that is involved. In support of the “dysregulated Th2” hypothesis, several human studies in Brazil where PPF was defined using ultrasound have indicated an important role for interleukin (IL)-13 in its development with elevated IL-13 levels in response to *S. mansoni* soluble egg antigen (SEA) observed amongst individuals with PPF (227–229). The association of Th2 cytokines with fibrosis and much of the pathology following *S. mansoni* infection has also been shown in murine studies of schistosomiasis (230–232). IL-13 is thought to induce its fibrogenic effects through the activation and production of transforming growth factor (TGF)-β (233), though TGF-β-independent mechanisms have also been suggested by studies using murine models (233). Experiments using the *S. mansoni* murine model have also shown that these type-2 immune responses are under epigenetic control with modulation of dendritic cells towards those that polarize T cells to type 2 (234), polarization of the T cells themselves (235), and alternative activation of macrophages (236) all being controlled by epigenetic modification. For macrophages, prior exposure to *S. mansoni* increased the expression of the demethylase that promoted alternative activation of macrophages (236), suggesting this response may be exacerbated when infection is prolonged.

Information from an autopsy study conducted in Ibadan, Nigeria also implicates a type-2 response in *S. haematobium* morbidity, though histologically a stage progression in lesion is found between children and adults (202). The early polypoid lesions, representing active infections in younger cadavers (mean age of 13-years), are characterized by loose granuloma formation around clusters of eggs with mass eosinophil infiltration, a cell type commonly associated with type 2 responses, particularly IL-5. In older individuals, lesions have more distinct mature granulomas, concentric circles of fibrosis and collagen deposition, more in line with the histological appearance of granulomas in the egg micro-injection model. When dissected these lesions have a “gritty” sensation, leading to the term “sandy-patch”. They appear to be a chronic manifestation often being associated with calcified eggs. A similar progression from loose eosinophilic lesions, in this case termed rubbery papules, to sandy-patches is observed in female genital schistosomiasis (237). Epidemiologically, leukocyturia, a symptom of *S. haematobium* infection, is highly correlated with egg counts (238, 239) and amongst a cross-sectional Sudanese cohort, 59% of individuals had eosinophiluria (defined as >=5% of urinary leukocytes). A disparity between urinary and circulating cell differential counts, with a mean of 42% of urinary cells being eosinophils, indicates that urinary eosinophils were tissue eosinophils shed into the urine. Eosinophiluria, but not other leukocyte counts or micro-hematuria prevalence, mirrored infection intensity (240), again emphasizing the role of the eosinophil within active *S. haematobium* bladder lesions. Eosinophil effector mechanisms include release of the toxic substances eosinophil cationic protein (ECP), eosinophil derived neurotoxin and major basic protein from granules. Urinary ECP levels in schoolchildren can differentiate between severity of bladder morbidity with a greater sensitivity than egg counts and as ECP levels are higher in infected schoolchildren without ultrasound detectable morbidity, than non-infected school-aged children, ECP is also a marker of early bladder wall inflammation (210). In addition ECP levels reflect resolution and re-emergence of bladder morbidity after treatment (210). Finally, ECP detection in vaginal lavage fluid is indicative of female genital schistosomiasis (FGS), with levels being higher for individuals with rubbery papules (241). The role of eosinophils in FGS is also indicated through decreasing vaginal lavage levels of ECP after treatment (242). In school-children low ECP levels prior to cell lysis, in comparison with potential cellular release as measured after lysis (243), suggest that eosinophils may not solely play traditional effector roles in response to *S. haematobium* eggs. Eosinophils are multi-functional immune cells, capable of antigen presentation and immune skewing and regulation by selective release of cytokines (244). Eosinophils are also a key component of the *S. mansoni* granuloma, and a significant association between a polymorphism within the ECP gene and presentation with PPF amongst the inhabitants of a Lake Albert fishing village has been observed, but this observation was dependent on the ethnic group of the human host (245).

In competing theory to the type-2 responses discussed above, a role for the pro-inflammatory cytokine tumor necrosis factor (TNF) in hepatic fibrosis and severe schistosomiasis disease in humans has been demonstrated by several studies. The first study to show a link between TNF (early studies do not clarify the member of the TNF family measured) and hepatic fibrosis was in Brazil (246). This was a hospital-based study and adult patients were categorized as hepatosplenic using clinical definition, rather than ultrasound. Further studies of community research subjects affirmed the association between elevated levels of TNF and increased risk of hepatosplenic disease (247–249). However, how the mechanisms behind PPF can differ between the sexes as reported in the Booth et al. study (247), with high TNF levels being associated with PPF in adult females but not males, is currently not clear. While the study of Mwatha and colleagues (248) was based on clinically rather than ultrasound classified school-aged children in Kambu region of Kenya, where a later study demonstrated hepatosplenomegaly in school-aged children in the absence of PPF (250). When immune responses of the children participating in the later study were examined, there was also high measurable TNF in response to SEA stimulation. When re-infection was abrogated through annual treatment and mollusciding of the river where transmission occurred, the TNF response to SEA diminished significantly (251). This raises the question as to whether TNF is a response to infection *per se* as opposed to part of the immuno-pathological disease process that leads to the development of PPF. We cannot know if the Kenyan children in the study of Mwatha and colleagues or the later study would have gone onto develop ultrasound detectable PPF. That said, studies in murine models have shown TNF to be an important mediator of granuloma formation and hepatic fibrosis (252), however, clear distinction between granuloma formation and PPF has to be drawn as we do not know how the two relate. Host genetic studies are contradictory regarding SNPs within the TNF loci; with no observable association between HLA-TNF polymorphisms and presentation with PPF in two Sudanese populations (253), but an association between a TNF gene SNP and PPF being reported for a Brazilian population (254). High TNF-alpha levels in response to SEA stimulation have also been shown to be associated with ultrasound detectable bladder morbidity due to *S. haematobium* infection in Kenyan case-control (255) and cross-sectional studies (256).

In stark contrast to the role for type-2 cytokines and TNF in fibrosis, community-based studies in Sudan (249) and Uganda (57) have associated high levels of interferon (IFN)-γ with reduced risk for fibrosis. In addition linkage between polymorphisms in loci closely linked to IFN-γ receptor genes (57) and in the IFN-γ gene itself (249) with PPF have been reported. Non-schistosome experimental murine studies support this anti-fibrotic activity of IFN-γ (257) and have associated it with modulatory effects of IFN-γ on TGF-β-induced immunopathology (258). Murine studies of schistosomiasis have associated IFN-γ with reduced infiltration of cells into the granulomatous sites and thus modulating the size of the granuloma (259). However, a further murine study of schistosomiasis has associated high IFN-γ levels with severe liver pathology during *S. mansoni* infection (260). In this study, mice that lacked IL-10 and IL-4 manifested mortality within few weeks of infection with *S. mansoni*, demonstrating that a balanced immune response, that is appropriately regulated, is what is required to limit pathology. In humans, *Schistosoma haematobium* infection has been shown to lead to hypermethylation of immune system genes within CD4+ T cells, with inhibition of genes in the Th1 and IFNγ signaling pathways being observed, indicating a role for epigenetic control of the IFNγ response. This inhibition was shown to persist 6-months after treatment (261).

Granuloma size in schistosome-infected animal models has been shown to peak between 6-9 weeks after infection, after which time they spontaneously regress in size (262, 263). In experiments where spleen, lymph node and T cells were adoptively transferred from chronically infected mice, granuloma size was shown to be reduced in the recipient animals (264, 265). From these experiments, it is apparent that the spleen and lymph node cells play an important role in the immunoregulation of granuloma formation. A role for B cells and their FcγR (IgG receptor) in the immunoregulation of the granuloma has been previously reported (266–268). Chronic schistosomiasis has also been associated with increased frequencies of FOXP3+ T regulatory (Treg) cells, and a role for Tregs in the control of morbidity during *S. mansoni* infection has been reported in both human and murine studies. A study of community members in Kisumu, Kenya reported increased frequencies of Treg cells in adults infected with *S. mansoni* (269) while treatment of *S. haematobium* infection amongst Gabonese children resulted in a decrease in Treg cells indicating that their numbers are supported during active infection (270). In another Kenyan study, the removal of Tregs from PBMCs donated by *S. mansoni*-infected individuals was associated with reduced levels of the key regulatory cytokine IL-10 (271), supporting the notion that Tregs are one of the sources of IL-10 and their immunoregulatory function is partly mediated by this cytokine. The removal of Tregs from SEA stimulated PBMC cultures of *S. haematobium* infected children increased the production of type-2 and pro-inflammatory cytokines (270). In a further study of *S. haematobium* infected school-children, B regulatory cells were shown to be important in inducing the expansion of IL-10 producing T cells (272). Booth et al. reported an association between low IL-10 scores following stimulation of blood with schistosome antigens and PPF in children from a community at Lake Albert in Uganda (247). While Kenyan study participants with high levels of TNF-alpha associated with bladder morbidity also had low levels of IL-10 (255, 256). Studies in murine models of schistosomiasis have supported these observations by demonstrating increased proportions of Tregs following infection of mice with *S. mansoni* or injection with SEA (273–276). The importance of Tregs in the control of immunopathology during *S. mansoni* infection has further been demonstrated by adoptive transfer of purified CD25-depleted T CD4^+^ T cells into mice without mature T and B cells. This depletion of CD25 CD4^+^ T cell (including Treg cells which express high levels of CD25) resulted in severe disease in these animals (275). The Tregs perform their immunoregulatory functions through IL-10-dependent and IL-10-independent mechanisms. The suppression of CD4^+^ T cell expansion and egg-induced Th1 responses by IL-10-producing Tregs has been reported in murine models of schistosomiasis (276). However, IL-10-independent mechanism of down-regulation of Th2 responses has also be reported (277).

Another aspect of the host that may be pertinent to the persistence of disease despite multiple rounds of treatment are variations in the pharmokinetics (PK) of PZQ; the subject of a recent systematic review (278). The authors of the review note that, whilst no pharmacogenetics studies have apparently yet been carried out for PZQ, SNPs in cytochrome P540 enzymes have been hypothesized to result in differing PK of PZQ. In the context of morbidity hotspots, differences in PK will not directly lead to development of morbidity, only indirectly acting through persistent infection, so parasite and other host factors will still be of the utmost importance. Regarding whether or not PK can change with increasing number of treatment rounds of PC impacting on its effectiveness, the authors of the systematic review make no note of acquired changes to the PK of PZQ. They do, however, raise the impact of liver morbidity on PZQ metabolism. The source paper (279) showed decreased metabolism of PZQ with increasing Child-Pugh scores of liver function (based on laboratory and clinical criteria, rather than ultrasound), though no difference in the cure rates between individuals with varying liver pathology were found. As ultrasound for PPF was not undertaken and a significant number of the cohort had positive makers for co-infection with viral hepatitis (85% for Hepatitis B and 99% for Hepatitis C), the impact of schistosomiasis mansoni morbidity on PK of PZQ remains largely unknown, but it certainly raises the question of whether co-infection with active viral hepatitis impacts on schistosomiasis treatment efficacy.

Overall, regarding host intrinsic factors, one may suspect that for genetic predisposition to result in a morbidity hotspot would require greater carriage of the predisposing profile within the population not just at the individual level. To date, human genetic studies have been conducted within populations, with some repeat association studies conducted in other populations, but to our knowledge no widescale genetics studies comparing susceptibility across populations has been conducted. We do know that within Lake Albert fishing communities that had high prevalence of PPF prior to MDA, that the Bugungu (ethnic-linguistically Bantu) and Alur (ethnic-linguistically Nilotic) people, despite differing ECP polymorphism linkage with morbidity (245), did not appear to have differing overall susceptibility to morbidity (33). This suggests no population level differences in susceptibility between these two ethnically diverse groups within this morbidity hotspot and perhaps indicates that genetic predisposition of the host may be more important in the manifest outcome of a morbidity hotspot i.e. who within the hotspot develops morbidity rather than being the underlying cause of the hotspot itself. The same would be true of genetic influences on PK of PZQ, with an influence on who within the population responds best to treatment, with greater potential for resolution of morbidity, rather than on the maintenance of the morbidity hotspot itself. Epigenetically, perhaps there could be some argument that past exposure/exposure of previous generations could predispose a population to mounting responses that are associated with the development of morbidity. However, contrary to supporting long-term epigenetic modifications passing from mother to child that result in increased morbidity within a population, murine *in utero* exposure to *S. mansoni* leads to a significant decrease in acetylation of the IL-4 promotor, reducing type-2 responsiveness of naive T cells of the offspring (280). There are not, to our knowledge, any published studies examining the potential relationship between epigenetics and schistosomiasis-associated morbidity in the human host.

## The Role of Host-Parasite-Environmental Interactions in Morbidity

From the combined knowledge derived from autopsy and epidemiological studies it is clear, for both *S. mansoni* and *S. haematobium* infections, that longevity of exposure to the egg antigens results in progression of fibrotic morbidity and that particularly amongst adults this can result in chronic, extensive fibrosis that is hard to treat through PZQ alone. It is also clear that key to the development of severe disease, particularly for PPF, is the breakdown of the regulation of the immune responses elicited against the egg antigens of the schistosomes. This regulation is imperative in protecting host tissue but also allows the parasite to sustain chronic infection by limiting damage to their host, and thus passage of its genetic material to future generations. It is therefore important to note that besides direct damage induced by the worms and their eggs, an aspect of helminths virulence is that they have evolved sophisticated mechanisms allowing them to evade or manipulate their hosts’ immune response and thus sustain a chronic infection. Helminths’ masterful manipulation of the immune system has mostly been characterized from murine experimental models of nematode infections and is mainly attributed to their secretomes and the variety of immunomodulatory products interacting with host tissues at every phase of the host immune response (281). This ability/inability to induce immunomodulation could thus be assimilated to virulence factors as defined in microparasites. As discussed, it is clear that regulatory immune cells and cytokines are induced during schistosome infection. Although not necessarily as well characterized as some of the immunomodulatory molecules secreted by nematodes, it is known that schistosome egg derived molecules, such as *S. mansoni* antigen IPSE/alpha-1, can induce expansion of regulatory B-cells, though this was concluded not to be the sole molecule within SEA that has this capability (276, 282). Although much work is needed to further characterize such factors in macroparasites in general and in particular for schistosomes, an interesting open question is to know whether such immunomodulatory molecules may vary at the parasite population level and across geographical zones, hence playing a role in persistent hotspots of infection and morbidity.

**Figure 1:**
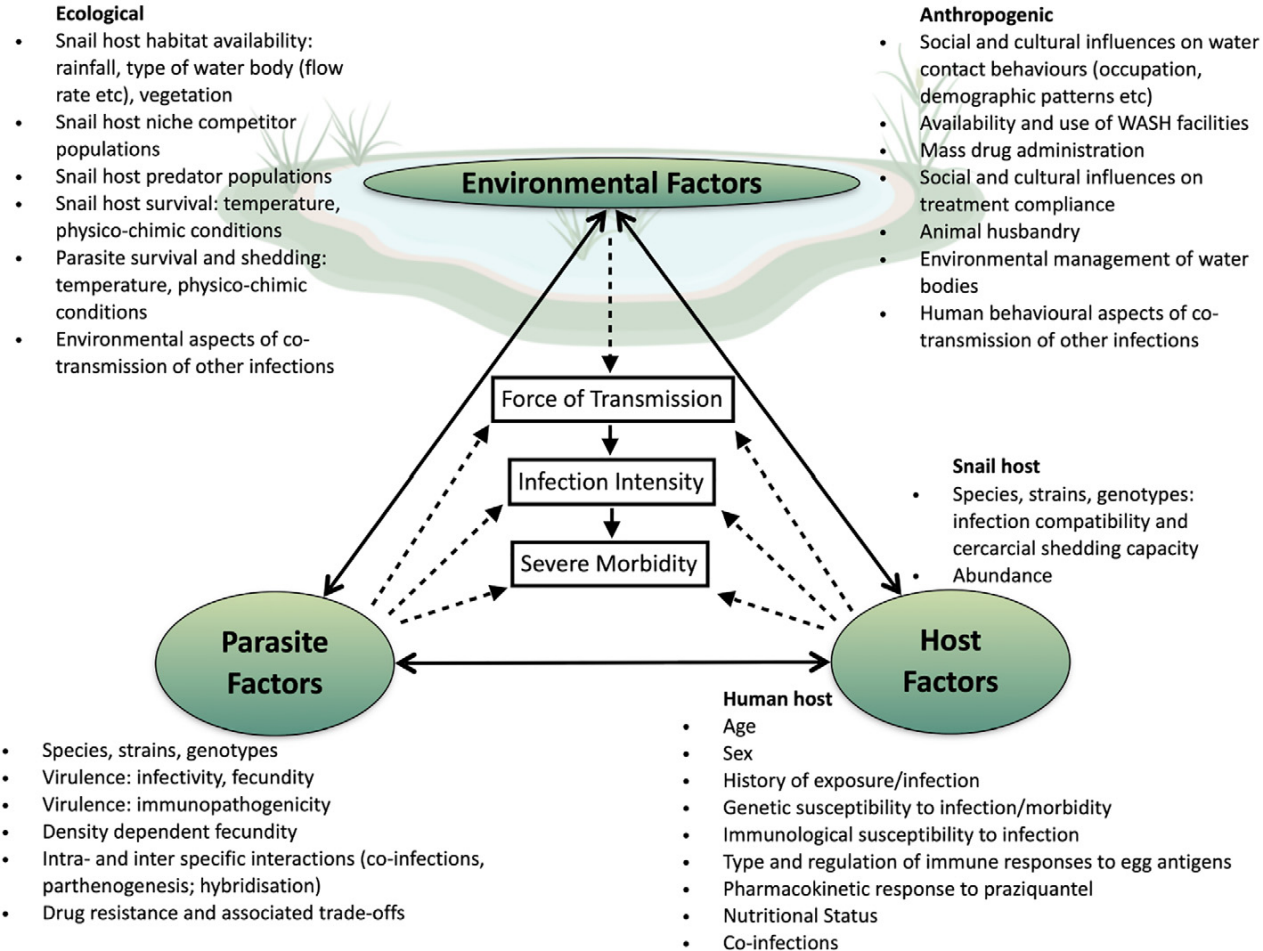
Potential drivers of persistent transmission and morbidity hotspots. Within the center of the transmission triangle are three elements known to be directly linked (solid arrows): force of transmission from the snail to human host is directly related to accumulation of infections over time and thus an increase in intensity of infection as measured by egg excretion; in turn high infection intensity is a known (but not sole) factor in the development of morbidity. Force of transmission of *Schistosoma* species is influenced by interactions (dotted arrows) between environmental, parasite and host factors. Once within the human host further interactions between parasite and host will determine the successful accumulative establishment of adult worm pairs and the fecundity of those worm pairs. Further interactions between the parasite and host will influence whether the host will develop severe morbidity. While interactions between environmental, parasite and host factors combine to drive up the force of transmission resulting in hotspots, these factors do not exist in isolation of each other, with direct influences from one corner of the transmission triangle to another occurring.

The complexity and multifactorial dimension of schistosomes transmission including host factors (behavior and water usage, genetics, age, sex and susceptibility, compliance to taking the drug); site-specific factors (sanitation, and type of water contact, or locations) as well as intermediate host factors (abundance, strain, species); but also differences in parasites factors (species and strains, parasites genotype and diversity, parasites ecology) and parasites interaction (co-infection, competition and hybridization), are all fundamental to our comprehension of schistosomiasis disparities in morbidity and persistent hotspots identified across the African continent (**Figure 1**). In these times of global changes and extensive human induced selective pressure, it is worthwhile considering that the parasite’s evolutionary history may be undergoing substantial modifications affecting their genetic diversity, transmission dynamics, virulence, and drug resistance development across Africa (59), with some potential implications for schistosomiasis clinical outcome and control. In this context, persistent biological hotspots and the failure of control strategies in several endemic areas is likely to be affected by host-parasite-environmental interactions and associated trade-offs such as increased virulence in parasites. To date we cannot rule out variable drug effectiveness, reduced efficacy and drug resistance, nor an increase in any specific genotypes/genotype combinations that are associated with increased virulence and persistent morbidity. Although such modifications can be monitored through the integration of clinical and parasitological measures with population genetic analyses using microsatellite markers, it is also important to point out that the small number of neutral genetic markers used in most studies and the fact that they do not span the entire genome may be poorly resolutive in comparison to whole-genome sequencing approaches. Therefore, if only a few alleles were to be involved in the parasite’s life history changes, there is a strong possibility that changes in allele frequency at the population level would not be detected by such methods. Indeed, although some parasite individuals may contain a few alleles responsible for resistance or other virulence related traits they may not cluster together because the majority of other alleles may vary at the individual level in the infrapopulation or component population. Furthermore, because cure rates are not 100% effective (85), it is worth considering that not only resistant parasite may be found after treatment, bringing some additional background noise into the genetic analyses. The continuous gene flow between human and animal parasite populations that undergo different selective pressures, in particular in relation to drug treatment, certainly also has an important role to play here in the persistence of these biological hotspots, together with intensive transmission and rapid re-infection of the hosts. It is thus important to implement a One-health framework in further control programs where humans at the community level (e.g., both children and adults), but also the parasites remaining in the (relatively) “drug free” environment (e.g., in snails, and in animals), would be targeted for reduction in schistosomiasis. The current lack of specific genetic markers for PZQ resistance and virulence, but also the fact that we mostly rely on neutral markers is a crux limitation in our ability to understand the role of parasite genetic variation in host disease phenotype in human schistosomiasis, including the wide-scale effect of drug treatment on parasite population genetic response. Thus, the development of more powerful non-neutral molecular markers (i.e., SNPs) at a genome wide level is warranted if we are willing to assess the strength of the genetic bottleneck after treatment, dissect re-infection from persistent infections, but also genetic changes that may be associated with the parasite’s fecundity, virulence and associated host induced morbidity.

Once SNPs are identified, elucidating their role thus confirming them as markers of virulence within the parasite population will only be possible if, at the same time, we obtain a greater understanding of the dysregulated immune response that leads to the development of liver PPF and severe bladder, upper urinary tract and genital tract fibrosis in humans. This includes the dissection of the similarities and differences observed in the immuno-pathology seen in *S. mansoni* and *S. haematobium* infections, as virulence factors may differ between the two species and indeed within species across their geographical range. Of note, the newly released WHO NTD Roadmap 2021 to 2030 (19), explicitly highlights the need for defined indicators of host morbidity. Without this linkage between parasite factors and host response, our certainty that we are monitoring for parasite SNPs that are indicative of potential resurgent hotspots of morbidity if the environmental factors are not controlled will be impacted.

## Case Study: Ugandan Albertine Region and the FibroScHot Project

Working with the Schistosomiasis Control Initiative, Uganda was at the forefront of establishment of the MDA-based control programmes within SSA with the first communities there being treated in 2003. These communities were based upon the shores of Lake Albert, an area that had historically been shown to have high infection intensities and a high prevalence of severe schistosomiasis (78, 172, 283). Success of the annual PC approach was established both empirically through measurements (172) and *via* anthropological investigations (284). However, in recent years reports of a high incidence of individuals presenting with portal hypertension at health clinics has indicated that severe schistosomiasis is currently prevalent within these areas (285). Given the age-association and long-term exposure that can lead to PPF, one explanation is that those presenting at the clinics with PPF are individuals who were not treated as children. However, high reported programme coverage rates from the districts indicate that the situation on Lake Albert is representative of a biological hotspot, where local factors in the ecology and host behavior of transmission have resulted in poor control of infection. Biological hotspots also occur in Ugandan communities resident in the fishing villages of Lake Victoria, with equally poor resolution of infection prevalence through the control programme (17). However, while those living on the shores of Lake Albert harbor high prevalence and strong intensities of infection with high rates of PPF (33, 35, 77–79, 283), in Lake Victoria communities, even prior to the implementation of the control programmes when infection levels were very high, observation of PPF was rare despite the infection levels of a comparable level to those in Lake Albert communities (36). These findings are indicative that not only is Lake Albert a biological hotspot of transmission, but that factors at the parasite-host interface lead to this being a morbidity hotspot. As PPF in children has been shown to generally be mild (277) and the observed association with infection intensities indicating that they are in an active phase of developing PPF there is some hope that the PPF observed can be reversed with PZQ treatment, but that the current annual administration is insufficient. From this the FibroScHot trial, which aims to determine whether treatment of schoolchildren twice or four times per year provides morbidity control was devised.

The primary objective of the trial at the center of the FibroScHot project is to compare the impact of twice and four times treatments annually with PZQ, relative to the standard once annual treatment, on the prevalence of *S. mansoni-*associated PPF of the liver. A secondary objective is to compare the effect of the same treatment strategies on the mean infection intensity of *S. mansoni*. In addition to the traditional statistical analysis of results, the consortium includes mathematical modelling expertise to allow impact of the strategies on a term longer than the 2-year duration of the trial to be examined. Predictive mathematical models will initially be built using historical data from the village of Booma for which behavioral aspects of human host exposure are well defined (286), though FibroScHot itself will be conducted in the neighboring district of Hoima. Data from the FibroScHot trial will be used to validate these models. Crucially, exploratory studies will run alongside the main clinical trial to determine any significant limitations to this solely PC approach to disease control. Medical anthropological analyses will examine the social and cultural influences on treatment uptake, while in context of this review both parasite and host factors will be studied. While we are cognizant of the potential role of co-infection, particularly viral hepatitis (B and C) and *Plasmodium*, in exacerbating morbidity within these communities, as the individual randomized trial design assumes co-infection will be comparable across the study arms, the study design is not optimal for research into this and it will not be explored further within FibroScHot.

The fact that PPF is more prevalent in Lake Albert region than the Lake Victoria region may be predicted to be due, at least in part, to parasite factors that prevent the successful down-regulation of immuno-pathological responses, which over time could lead to expansive fibrosis. The FibroScHot study aims to make some progress on the above by integrating examination of the childhood responses to schistosome egg stimulation with examination of parasite population genetics and genome sequences. As discussed in the main article, due to the epidemiological patterns of PPF, many human studies that have investigated immuno-pathological mechanisms of PPF report results biased towards the responses observed amongst adults. Exploring the immune responses of children in the active phase of developing PPF could give us insight into the mechanisms that drive the development of severe schistosomiasis. The childhood PPF observed in Lake Albert region of Uganda gives us an opportunity to do this. While the project is not powered to provide clear evidence of “pathogenicity” related regions within the parasites, it will allow us to identify changes in parasite populations through time. Examining gene flow within the parasite population will identify specific isolates associated with reduced egg reduction rates or persistent morbidity. This population genetics approach will be combined with genome wide sequencing of selected parasite isolates, allowing us to elucidate both potential indicators of reduced PZQ efficacy and/or specific novel genotypes relating to increased PPF within these populations. This will constitute the initial steps required for fully integrated research into the interactions between parasite and host factors that may contribute to Lake Albert being a morbidity hotspot.

## Conclusions

Schistosomiasis is a multifactorial disease in which several host and parasite factors including their interaction with the environment may act at each step of the parasite’s life cycle (287, 288). While the reasons behind discrepancies in clinical outcome remain ambiguous and the relationship between infection intensity and organ-related morbidity is complex, cumulative evidence is now showing that as well as host-specific factors shaping the genetic composition of schistosome populations (156, 190, 191), parasite genetic differences may indeed be important in the development of human host morbidity (53, 133, 175, 176, 289), and the characteristics of morbidity responses to them (150). Regardless of whether or not morbidity hotspots are driven by intrinsic host factors, parasite factors, or a combination of both in complex interplay with the environment, the fact that these hotspots exist does mean that the use of infection parameters to define morbidity control, and in the context of the new Roadmap, elimination of schistosomiasis as a public health problem in all 78 endemic countries by 2030, is likely insufficient. Given particularly that severe morbidity in these hotspots may develop at lower infection levels, or in a greater proportion of individuals, if the host-parasite relationship allows, the current use of an infection parameter alone to define success could well be misleading, leaving a significant number of individuals in further need without appropriate treatment. There are of course financial and logistical problems to overcome if morbidity screenings, in the absence of easily measurable markers of morbidity, were to become routine in control programmes, not least the requirement for the skilled personnel to conduct the ultrasonographic investigations. One approach would be for morbidity screens to be integrated into the verification of elimination as a public health problem within countries. Ultimately, understanding how the morbidity profiles of schistosomiasis differ and change across different endemic areas will prove to be of ever-increasing critical importance to the monitoring and evaluation of control programs in reaching the goal of elimination of schistosomiasis as a public health problem in all endemic countries.

## Author Contributions

PM and JK-S wrote the first draft of the article, with subsequent input from EMT, SW and JW. All authors contributed to the article and approved the submitted version.

## Funding

All authors were supported through the FibroScHot project which is part of the EDCTP2 programme supported by the European Union (RIA2017NIM-1842-FibroScHot).

## Conflict of Interest

The authors declare that the research was conducted in the absence of any commercial or financial relationships that could be construed as a potential conflict of interest.
